# Editorial for the Special Issue on Micromachined Acoustic Transducers for Audio-Frequency Range

**DOI:** 10.3390/mi16010067

**Published:** 2025-01-08

**Authors:** Libor Rufer, Shubham Shubham, Haoran Wang, Tom Miller, Petr Honzík, Vittorio Ferrari

**Affiliations:** 1 Advanced Design & Technology for MEMS, 38140 Rives, France; 2 Syntiant Corporation, Itasca, IL 60143, USA; shubham.shubham@syntiant.com; 3 Applied Materials Inc., Santa Clara, CA 95054, USA; wanghaoranuf@gmail.com; 4 Knowles, Electronics LLC, Itasca, IL 60143, USA; tom.miller@knowles.com; 5 Department of Radioelectronics, Faculty of Electrical Engineering, Czech Technical University in Prague, 166 27 Prague, Czech Republic; honzikp@fel.cvut.cz; 6 Department of Information Engineering, University of Brescia, 25121 Brescia, Italy; vittorio.ferrari@unibs.it

## 1. Introduction

Microelectromechanical systems (MEMS) refer to miniaturized mechanical and electro-mechanical elements that are fabricated through microelectronic processes. The development of MEMS technology dates back to the 1960s, along with the development of silicon-based integrated circuits and advancements in micromachining techniques. Early MEMS research primarily focused on sensors and actuators, where microfabrication methods such as photolithography, etching, and deposition enabled the creation of highly precise structures at microscopic dimensions.

In the 1980s and 1990s, MEMS technology gained traction as advances in semiconductor fabrication processes made it possible to manufacture complex structures at a very small scale. These developments enabled MEMS devices to operate with high accuracy and low power consumption, sparking innovations across a range of industries, including automotive, biomedical, telecommunications, and consumer electronics.

One of the most significant and impactful applications of MEMS technology is in the field of acoustics. MEMS-based acoustic devices, such as microphones, speakers, and sensors, have revolutionized the way sound is captured, processed, and reproduced. MEMS microphones, for example, leverage the small size, low power requirements, and high sensitivity of MEMS technology to create miniature microphones that are widely used in smartphones, hearing aids, and other portable devices. These microphones provide high performance and durability in compact packages, making them ideal for consumer electronics that demand miniaturization without degrading the main parameters. The integration of MEMS technology in acoustic applications has also led to innovations in noise-cancelling headphones, smart speakers, and acoustic sensing systems for industrial and medical purposes.

The future of MEMS in acoustics looks promising, with ongoing research pushing the boundaries of device performance, miniaturization, and integration. As this technology continues to evolve, we can expect further innovations in audio processing, smart devices, and immersive acoustic experiences, transforming industries ranging from entertainment to healthcare. The integration of MEMS with the internet of things (IoT) will accelerate this transformation, enabling smarter, interconnected devices that can seamlessly communicate and adapt to their environments. MEMS-based acoustic sensors in IoT applications, for example, will enhance real-time audio analysis, environmental monitoring, and personal health tracking, leading to more efficient, responsive, and intelligent systems.

This Special Issue featured a total of ten contributions, comprising nine original research articles and one review paper. Each contribution integrates one of the main topics in the field, which are detailed in the following paragraphs.

## 2. MEMS Microphones

### 2.1. Overview of Recent Developments

MEMS microphones are widely used in various applications, including IoT devices such as smartphones, speakers, tablets, laptops, smartwatches, and true wireless earphones, as well as other consumer appliances in automobiles, industrial goods, and machinery. Due to their miniaturized size, low cost, high-quality acoustic performance, exceptional integration compatibility with application-specific integrated circuits (ASICs), and improved reliability against environmental factors, there has been a continuous demand to further improve the signal-to-noise ratio (SNR) performance and other key parameters related to MEMS microphones, including sensitivity, noise, total harmonic distortion (THD), and dynamic range.

The authors of [1] presented a new differential MEMS capacitive microphone transducer design based on a sealed-dual membrane (SDM); they significantly reduced the fluidic noise component caused by air inside the MEMS transducer by eliminating the air between the two membranes. An SNR of 72 dBA was reported, with a sensitivity of −38 dBFS and an acoustic overload point (AOP) of 130 dB; this was achieved with latest generation digital ASIC and a reduced power consumption. This SNR performance marked a significant improvement on the current state-of-the-art MEMS microphones on the market, which have an SNR in the range of 65 to 70 dBA [2,3,4]. In 2021, a single corrugated membrane design with a single-backplate architecture and capacitive transduction mechanism was proposed, delivering a 73 dBA SNR with an analog ASIC, closely matching the performance of the SDM MEMS with a localized vacuum [5]. Again, the focus was on minimizing the MEMS acoustic damping losses to achieve a high SNR performance. The MEMS sensitivity achieved was −34 dBV/Pa. Steady progress has also been made in the piezoelectric transduction mechanism, which offers the advantages of low power consumption and makes the sensor less susceptible to particle contamination. In 2023, a novel design based on a fully clamped corrugated aluminum nitride (AlN) membrane was presented, offering very effective intrinsic stress relaxation and enabling differential signal generation by exploiting a pseudo-bimorph effect [6]. A sensitivity of −47.1 dBV/Pa and an SNR of 62.6 dBA were reported. However, the authors left room for further design improvements to achieve higher mechanical compliance, while maintaining a system resonance above 20 kHz, and enhance the overall performance. To address the impending fundamental physical, design, and material limitations for MEMS microphones, several works have demonstrated the use of graphene as a diaphragm. In 2024, a novel approach to integrating multi-layer graphene into condenser MEMS microphones, without the need for transfer or polymer support, was presented [7]. This approach addressed the previous limitations associated with fabricating wafer-scale graphene microphones without polymer supports, potentially opening new possibilities for future microphone technology. The sensitivity achieved was 24.3–321 mV/Pa, which is more than twice that of the reported state-of-the-art MEMS microphones. Further exploration is needed to enhance other critical MEMS microphone performance metrics. On the microphone modeling front, a behavioral model to capture the nonlinear phenomena in MEMS microphones was developed using a circuit simulator tool and hardware description languages (Verilog-A) [8]. The proposed model improved the accuracy of predicting sensitivity, SNR, and THD performance. A constrained MEMS microphone architecture was used to validate the findings with measurements. Shubham et al. [9] also demonstrated a similar behavioral model for capturing nonlinearities using circuit modeling tools to represent blocks with couplings between the acoustic, mechanical, and electrical domains. Although the approach and intent were similar, the modeling method presented was generalized to cover devices for which there were no closed-form solutions regarding the diaphragm shape or variable capacitance of the microphone. This became increasingly important when considering the boundary conditions of the diaphragm and its deformed shape under applied bias or acoustic pressure. These models excel in precisely predicting pull-in, sensitivity, SNR, and THD performances over wide acoustic pressure ranges.

### 2.2. Future Research Areas to Address Knowledge Gaps in the Field

There has been a continuous push to expand the boundaries of physics in order to further improve the SNR performance, enhance the sensitivity, and reduce the overall noise of MEMS microphones. This will require focusing on minimizing other components, such as noise related to the thermal boundary layer. The noise floor in the MEMS microphone design is limited by the thermal noise generated by the heat exchange with the isothermal boundary condition at the walls in the back cavity. This typically results in increased noise as the microphone package size decreases. A diffusion-dominated design regime has been explored that can yield high SNR with very small back volumes. In such a design, the challenge becomes producing enough output signal, which often requires the use of very-high-bias voltages in the sensor [10,11]. The need for good MEMS microphone performance has opened the door for the further exploration of other transduction mechanisms, such as optical technology. Although the proof-of-concept dates back to 2003, it was not until 2016 that a Norwegian company, SensiBel, demonstrated a breakthrough with an 80 dBA microphone SNR and 146 dB AOP using this technology [12]. However, the typical challenges related to power consumption, size, and the high cost of optical components remain unresolved. To overcome the design challenges and improve the performance of MEMS microphones, this Special Issue also included novel design features. Shubham et al. [13] proposed a semi-constrained diaphragm design with flexible springs, supported by center and peripheral protrusions originating from the back plate. This helped to significantly increase the effective area of the diaphragm by 85% under bias, thereby improving the linearity, sensitivity, and SNR performance of the microphone.

All the modeling explorations assume a rigid (clamped at all boundaries) perforated backplate. However, in this Special Issue, Šimonová and Honzík address this gap by including the movement effect of the perforated moving electrode to accurately capture the damping effects related to thermal and viscous boundary layers; they compare the analytical results with finite element modeling [14]. In another study [15], the authors considered the backplate as a vibrating structure and extended the lumped model to introduce coupling between the mechanical and electrical domains, showcasing the impact on the microphone sensitivity and device pull-in. In this Special Issue, Rufer et al. address concerns related to the poor AOP performance of MEMS microphones under high sound pressure levels and in harsh environmental conditions [16]. A novel approach to improving the AOP performance is through piezoelectric coupling. With a modeling and optimization approach for microphone sensitivity and AOP tuning in the audio band, the piezoelectric MEMS microphone can be used in applications that require high sound pressure level detection, such as aircraft design, rocket-launching vehicles, gunshot detection, and urban security systems. The issue also demonstrates a new approach to enabling sensitivity control by placing one electrode at the diaphragm center and one annular electrode closer to the clamped edge of the diaphragm. The first electrode serves as a sensor to provide the output signal, whereas the second acts as an actuator that mechanically pre-stresses the diaphragm and alters the microphone sensitivity.

## 3. MEMS Speakers

In the past few years, MEMS speakers, another type of micromachined acoustic transducer, have drawn significant interest from researchers and businesses. With the flourishing development of consumer electronics and artificial intelligence (AI), there are increasing demands for miniaturized speakers in headsets, smartphones, hearing aids, voice assistants, and internet of things devices. Benefitting from micromachining technologies, MEMS speakers offer numerous advantages; they have a small carbon footprint, a light weight, low power consumption, low-cost batch fabrication compatibility, and an improved compatibility with application-specific integrated circuits.

However, as a consequence of their small device size, most of the existing MEMS speakers face the challenge of limited sound pressure output [17]. Sound quality and linearity in frequency response, which are typically evaluated according to the total harmonic distortion (THD), are also important considerations in the development of MEMS speakers [18]. Significant progress has been made in addressing challenges related to materials, device structure designs, electrode configuration, and driving methods [19,20]. Lumped element modeling and acoustic simulation are also important methods that have been explored to effectively optimize MEMS speaker design and performance [21].

According to transduction mechanisms, MEMS speakers are classified as electrodynamic, electrostatic, piezoelectric, and thermoacoustic. Breakthroughs have been made in all types of MEMS speakers, including in terms of the various materials and fabrication approaches, special device designs, and improved sound pressure levels (SPLs) over wide bandwidths [22,23]. Due to their ability to achieve large actuation forces with relatively small driving voltages, piezoelectric MEMS speakers show the most promising results and have become a research hotspot. In terms of recent developments, there are more and more studies dedicated to piezoelectric MEMS speakers.

In this Special Issue, we include three such studies. In particular, Wang et al. presented a comprehensive review of MEMS speakers, including their recent development, remaining challenges, and future outlook [24]. Liechti et al. proposed a method to evaluate the THD generated by a cantilever-based MEMS speaker inside an acoustic coupler [25]. Teuschel et al. investigated the potential of using epitaxial grown PZT with an imprint for stable bipolar operation, demonstrating a strong piezoelectric response for MEMS actuator applications such as speakers [26].

In the future, more research is expected on how to improve the SPL of MEMS speakers. Obtaining large driving forces with low power consumption will continue to be a key developmental challenge. A balance between small diaphragm sizes and relatively large SPL outputs can be achieved by exploring new materials, novel structure designs, and special electrode configurations. With a more comprehensive understanding of the modeling, special structures and acoustic enclosures will also be explored in order to optimize the overall frequency response of MEMS speakers, including further reduced THD and high SPL over wide bandwidths. In addition to the optimized device performance, easier fabrication techniques with low costs, high yields, and good compatibility with other materials and processes will continue to be studied before MEMS speakers can become mainstream and large-scale commercial products.

## 4. MEMS in Hearing Aids and Implantable Devices

MEMS microphones first saw mass market acceptance with their introduction into cell phones in 2003 and have been dominant in hearing health products since 2019. They offer improved electroacoustic performance, reliability and repeatability at a lower cost than electret microphones. Devices currently offer noise levels below 25 dBA, handling sound levels over 135 dB in a package less than 3.5 mm long [27]. Knowles Electronics pioneered the creation of MEMS microphones for the hearing health and commercial markets and continues to be a key supplier. Sonion is now also a significant supplier to the hearing health market. InvenSense is a key supplier of MEMS silicon to many makers of MEMS-based microphones. The latter face continued pressure to provide ever greater dynamic range at both the softest and loudest extremes while further reducing size. Future research goals include further reducing nonlinearity, greater resistance to contamination from exposure to dust and liquids, and selectively reducing sensitivity to ultrasound signals emitted by proximity sensors. Research in the ASICs for these products is seeking to reduce noise and distortion, reduce power consumption, and enable a growing list of programmable options such as gain, filtering, and power consumption.

Commercial applications of MEMS microphones are virtually all air-conduction. There has been research into using implanted sensors as microphones, with particular interest in their use with cochlear implants. However, their SNR and directivity are inherently much poorer than in air-conduction devices, preventing their widespread adoption for many hearing health applications. There is also potential for using implanted MEMS audio devices to monitor voice production and other audio signals within the body. One major hurdle to using MEMS implanted devices is protecting the delicate sensor from contact with bodily tissues and fluids while avoiding attenuating the signals the sensor should be receiving. In this Special Issue, Prochazka et al. describe a method for such protection that offers a reduced attenuation of the desired signal [28].

MEMS audio-frequency vibration-sensing devices attached to the outside of the body, particularly near the jaw and ears, are seeing increasing adoption. These are often referred to as bone-conduction sensors and the sensors are placed on the skin over areas of bone or cartilage that offer strong signals. Audio band vibration sensors can also be used to convert any vibrating surface in a room or on a car into a microphone [29,30]. Human body applications include speaker identification and speech capture augmentation in high wind conditions, for which high SNR and low power consumption are critical.

While body-contact MEMS sensors are in development, they do not yet make very effective actuators. The most significant issue is the very large impedance mismatch between the MEMS actuator and body tissues. The actuator must also be protected from the forces applied by the tissues. The transducers currently used in bone-conduction speakers use a moving mass that is many orders of magnitude larger than could fit into a MEMS device. It will be interesting to see if there are niche applications where a MEMS actuator could be useful. One possibility would be applying force directly to the tiny bones of the middle ear or to the eardrum.

MEMS speakers are beginning to be used in earphones, but have not yet seen acceptance in hearing health devices. MEMS devices struggle to produce the desired 120 dB sound levels in the ear canal. They also need high-voltage electronics to power them and hence significantly more battery power than traditional balanced-armature (BA) devices. This prevents them from meeting hearing aid designers’ battery life requirements. For comparison, the Knowles balanced armature driver package size is 5.3 × 3.1 × 2.7 mm, a size that is widely used in hearing instruments. This device can produce a sound level of 130 dB at its 3 kHz resonance and produces 112 dB at 1 mW of drive power (rising to 122 dB/mW at 3 kHz) [31]. The size of the BA device also includes the air enclosure behind the transducer. In hearing health applications, the sound radiating from the rear of the transducer must be contained in a sound-proof enclosure, so that it cannot leak to the microphone and cause feedback. MEMS speakers often require a high-compliance acoustic load behind the radiator, which can be larger than the BA housing.

## 5. Measurement Methods for MEMS Devices

Although most MEMS acoustic devices currently in use operate on the same principles and using the same methods as their traditional millimeter-scale counterparts, applying the same evaluation methods as those used for traditional devices is not straightforward. This paragraph highlights the extraction of MEMS parameters presented in this Special Issue, and provides examples of two additional methods specifically tailored for MEMS microphone characterization.

### 5.1. Extraction of MEMS Transducer Mechanical Properties

Extracting the mechanical properties of complex three-dimensional MEMS transducers through acoustic transmission measurements offers a novel approach that bypasses the limitations of traditional characterization methods. Becker et al. focus on folded diaphragms with buried in-plane vibrating structures, which expand the active area while maintaining a compact form factor [32]. Conventional techniques such as bulge tests and atomic force microscopy often require destructive preparation or rely on optical accessibility, which is infeasible for structures hidden within high-aspect-ratio trenches [33,34,35,36]. The presented methodology employs a passive acoustic transmission setup where diaphragms are actuated by an acoustic signal and analyzed using lumped-element modeling (LEM). This non-invasive method effectively captures dynamic mechanical behaviors such as compliance, even for complex geometries, without altering boundary conditions.

The experimental framework utilizes two acoustically isolated chambers separated by the diaphragm under test. Sound pressures on both sides of the diaphragm are measured to extract mechanical properties by fitting experimental data to LEM simulations [37,38]. The results demonstrate high accuracy, with the measured compliances differing by less than 4% from numerical simulations for diaphragms exceeding 1 mm in length.

By eliminating extensive sample preparation and ensuring boundary conditions mimic practical applications, this method advances the characterization of MEMS transducers with complex three-dimensional geometries. It provides valuable insights into the mechanical properties critical for optimizing the design and performance of MEMS devices in acoustic applications.

### 5.2. High-Frequency Calibration of MEMS Microphones

Due to their compact dimensions, MEMS microphones can achieve frequency responses extending into several hundreds of kilohertz. Characterizing the frequency response of such microphones and pressure sensors presents a significant challenge. To address this, a calibration method has been developed utilizing a spherical weak shock acoustic pulse generated by a spark source. The pressure waves, in the form of asymmetric N-waves with a duration of approximately 40 µs and a front shock rise time of around 0.1 µs, were characterized using an optical interferometer. By accounting for the nonlinear propagation of weak shockwaves, it became possible to estimate the incident pressure wave and derive the frequency response of MEMS microphones across a range of 10 kHz to 1 MHz [39,40]. This approach is independent of the transduction principle or sensor mounting configuration.

### 5.3. Measurement of MEMS Microphone Nonlinearities

Measuring the nonlinearities of MEMS microphones has become an important research area, particularly due to the widespread use of these microphones in consumer electronics and measurement applications. MEMS microphones are popular for their small size, low cost, high sensitivity, low inherent noise, and low power consumption. However, nonlinear distortions, such as harmonic and intermodulation distortions, can impact precision, making their investigation crucial. These distortions from multiple factors, including electrostatic transduction, the mechanical properties of the diaphragm, air gap damping, and amplifier behavior [41,42]. In particular, the electrostatic transduction mechanism is often identified as the primary source of distortion in condenser microphones, producing both harmonic and intermodulation distortion [43,44,45]. According to [46], the second harmonic contributes approximately 90% of the total harmonic distortion.

In a typical experimental setup used to study MEMS microphone nonlinearities, a reference low-distortion microphone and a loudspeaker can be employed to generate a sinusoidal acoustic pressure signal with minimized higher harmonics. Achieving a perfectly linear acoustic signal source is a challenging task, as most sources inherently introduce some degree of distortion. Therefore, harmonic correction [47] is advantageous in effectively reducing these distortions. The distortion of the nonlinear source (loudspeaker) is reduced to the noise level using this adaptive correction method, assuming that the nonlinearities of the reference microphone are negligible. This setup allows for a direct measurement of the harmonic distortion of MEMS microphones under testing at different frequencies and sound pressure levels. For example, the authors of [48] describe such measurements conducted at frequencies of 20 Hz, 200 Hz, and 2 kHz, and sound pressure levels ranging from 90 to 128 dB. A simple nonlinear model based on the electrostatic transduction principle was used to compare the measured results for single-backplate condenser MEMS microphones. The results indicate that the second harmonic behaves predictably and is well described by the model. However, the third harmonic distortion product shows deviations, suggesting the influence of nonlinearities not included in the simple analytical description.

To conclude, measuring the nonlinearities of MEMS microphones is feasible, especially with the use of advanced harmonic correction techniques that minimize source distortion. The electrostatic transduction mechanism is identified as the primary source of distortion, mainly affecting the second harmonic. However, challenges remain, particularly in modeling and accounting for higher-order nonlinear effects and other sources of distortion, such as diaphragm deflection and dynamic changes in air gap thickness [49]. Future work should focus on two main areas: refining models to include these additional nonlinearities for better prediction accuracy, and achieving effective reduction in nonlinear distortion, particularly by minimizing the level of the second harmonic in single-backplate condenser MEMS microphones.

Piezoelectric MEMS microphones can exhibit nonlinearities due to material properties and fabrication imperfections. The former occur when piezoelectric materials like aluminum nitride (AlN) respond nonlinearly under high electric fields or mechanical stresses, leading to harmonic distortion in the output signal [50]. Geometric nonlinearities arise from fabrication-induced imperfections, such as initial curvature or residual stresses in the diaphragm; these result in hardening or softening behaviors that affect device performance [51]. Understanding and mitigating these nonlinearities is crucial for the accurate and reliable operation of piezoelectric MEMS microphones, particularly in applications requiring high fidelity and precision.

## 6. Applications of MEMS Acoustic Devices

MEMS devices’ small size, low power consumption, ability to integrate with sophisticated electronics, and cost-effective mass production enable their widespread application across industrial, military, and consumer sectors. This paragraph presents two papers featured in this Special Issue, along with additional papers, illustrating some of their potential applications.

### 6.1. Acoustic Source Localization and Ranging

Acoustic source localization, ranging, and imaging are essential techniques for determining the position and characteristics of sound sources in various environments. Acoustic source localization involves pinpointing the location of a sound source by analyzing the time-of-arrival (TOA) or phase shifts of sound waves reaching multiple microphones or sensors. Since the distance is the product of the signal speed and the time of flight of the signal traveling from the source, the ranging accuracy depends on the signal speed and the precision of TOA measurements. Compared to other signal types, such as light or radio, acoustic signals are often better suited for achieving high accuracy due to their relatively slow speed. However, ensuring precise TOA measurements remains a significant challenge in system implementation [52].

Acoustic source localization is widely used in underwater sonar systems and applications such as speech tracking and wildlife monitoring [53]. Another important area of research is gunshot acoustic localization, which is employed in military and civilian security systems. In this context, microphone array data are used to identify the event time, the direction of arrival (DOA), and to calculate the shooter’s position [54].

Acoustic ranging is a technique for estimating the distance between two objects. It plays a critical role in applications such as motion tracking, gesture and activity recognition, and indoor localization. Although numerous ranging algorithms have been developed, their performance often degrades significantly under conditions of strong noise, interference, and hardware limitations. Various ranging applications using MEMS microphones have been explored, spanning simple methods with three sensors for sound source localization [55], gesture recognition [56,57], and indoor positioning systems for public use in smart city-related applications [58].

### 6.2. MEMS Microphone Arrays for Acoustic Imaging

Acoustic imaging takes acoustic source localization and ranging a step further, creating a spatial map or visual representation of sound waves as they interact with objects. This technique is widely used in medical imaging and non-destructive testing. Acoustic imaging is crucial in fields like marine biology, seismic exploration, autonomous vehicle navigation, and aeroacoustics, providing vital information about the environment through sound. Due to their small size, MEMS microphones are inherently well suited for integration into compact arrays, enabling high spatial resolution and controlled directional sensitivity in detecting acoustic fields [59]. In particular, aeroacoustic testing is an application where MEMS microphone arrays are both attractive and promising [60,61].

In this context, in this Special Issue, Ahlefeldt et al. report the results of an experimental study demonstrating that MEMS microphones assembled into an array allowed for detecting a plane fuselage’s surface pressure fluctuations during flight [62]. While the state-of-the-art sensors for measuring such fluctuating pressure distributions are small piezoresistive pressure transducers, MEMS devices offer smaller dimensions and reduced costs. The article describes testing MEMS microphones for in-flow measurements. Commercially available MEMS microphones have a limited AOP of up to a maximum of 135 dB, which is too low for in-flow operation. To overcome this limitation, a covering Kapton foil of 25 µm in thickness was applied on the microphones to attenuate the pressure by approximately 38 dB. The cover layer also introduces a change in the frequency response, which was taken into account. The results of both wind-tunnel and flight tests indicate that the Kapton covering damped the pressure fluctuations as expected, while still preserving the critical phase information required for wavenumber analysis.

### 6.3. Electrical Tuning of MEMS Acoustic Transducer Resonant Frequency

MEMS acoustic transducers can function as sound receivers (microphones) or emitters (speakers) within the audio-frequency range. Additionally, they can operate in the ultrasonic frequency range as micromachined ultrasonic transducers (MUTs), which are further categorized into piezoelectric (PMUTs) and capacitive (CMUTs) types.

The acoustic response and performances of the transducers depend, among other parameters, on the mechanical conditions in the microstructure; these conditions can affect its elastic properties, such as the degree of static stress in vibrating diaphragms and plates. Static stress can be influenced or determined by DC bias voltages purposely applied to suitable electrodes, particularly with piezoelectric MEMS transducers. This opens the possibility for the electrical adjustment/tuning of transducer properties relevant to acoustic response, such as resonant frequencies and corresponding quality factors. This approach has been investigated in the ultrasonic frequency range in PMUTs [63].

In such a scenario, in this Special Issue, Nastro et al. report the results of an experiment exploring and validating a technique to obtain an electrically tunable matching between the series and parallel resonant frequencies of a piezoelectric MEMS acoustic transducer [64]. This can increase the effectiveness of acoustic emission/detection in voltage-mode driving and sensing.

The adopted piezoelectric MEMS transducer is based on an aluminum nitride (AlN) active layer on top of a square diaphragm. By applying an adjustable DC bias voltage between pairs of properly connected electrodes, a planar static compressive or tensile stress in the diaphragm is electrically induced, depending on the voltage sign, thereby shifting its resonant frequency. The piezoelectric MEMS transducer operates at its first flexural resonance of about 5 kHz. The results of the experimental tests run in both receiver and transmitter modes showed that the resonance can be electrically tuned in the bias voltage range between −8 V and +8 V with estimated tuning sensitivities of about 9 Hz/V and 8 Hz/V in transmitter and receiver modes, respectively. The reported device can be employed in pulsed-echo mode as a proximity/presence or gesture detector. The proposed technique can be transferred to down-scaled structures to obtain tunable PMUTs.

A similar concept for controlling microphone sensitivity is presented in another paper in this Special Issue, that by Rufer et al. [16]. The proposed approach utilizes a MEMS piezoelectric microphone design featuring two electrodes: a circular electrode positioned at the diaphragm’s center and an annular electrode located near its clamped edge. One electrode operates as a sensor, generating the microphone’s output signal, while the other serves as an actuator. By applying a DC bias voltage to the actuator, the diaphragm can be mechanically pre-stressed, thereby altering the microphone’s sensitivity. This sensitivity control approach holds significant potential for applications involving high acoustic loads, where reduced microphone sensitivity can be electronically achieved when the acoustic level exceeds a predefined threshold.
